# Venous Stent Migration into Right Ventricle

**DOI:** 10.7759/cureus.1583

**Published:** 2017-08-19

**Authors:** Menfil Orellana-Barrios, Nachiket Patel, Aliakbar Arvandi, Ralph Paone, Dixon Santana

**Affiliations:** 1 Division of Cardiology, TTUHSC; 2 University of Arizona at Phoenix, Banner University Medical Center Phoenix; 3 Cardiothoracic Surgery, UMC Lubbock; 4 Vascular Surgery, TTUHSC

**Keywords:** venous stenting

## Abstract

Venous stents (VS) are used to treat central and peripheral stenoses. Stent embolization into a cardiac chamber is a rare, yet serious complication. We present a case of a 61-year-old man with a recently stented arteriovenous graft venous stenosis who developed VS migration into the right ventricle, associated with S. aureus bacteremia.

## Introduction

Venous stents (VS) are used to treat stenoses in central and peripheral veins. These stenoses can occur due to vein injury, inflammation, turbulent flow and uremia. Venous stenoses are particularly problematic when they involve hemodialysis access in patient with end-stage renal disease. We present a case of a 61-year-old man with a recently stented arteriovenous graft (AVG) venous stenosis who underwent VS placement with subsequent related complications.

## Case presentation

**Figure 1:**
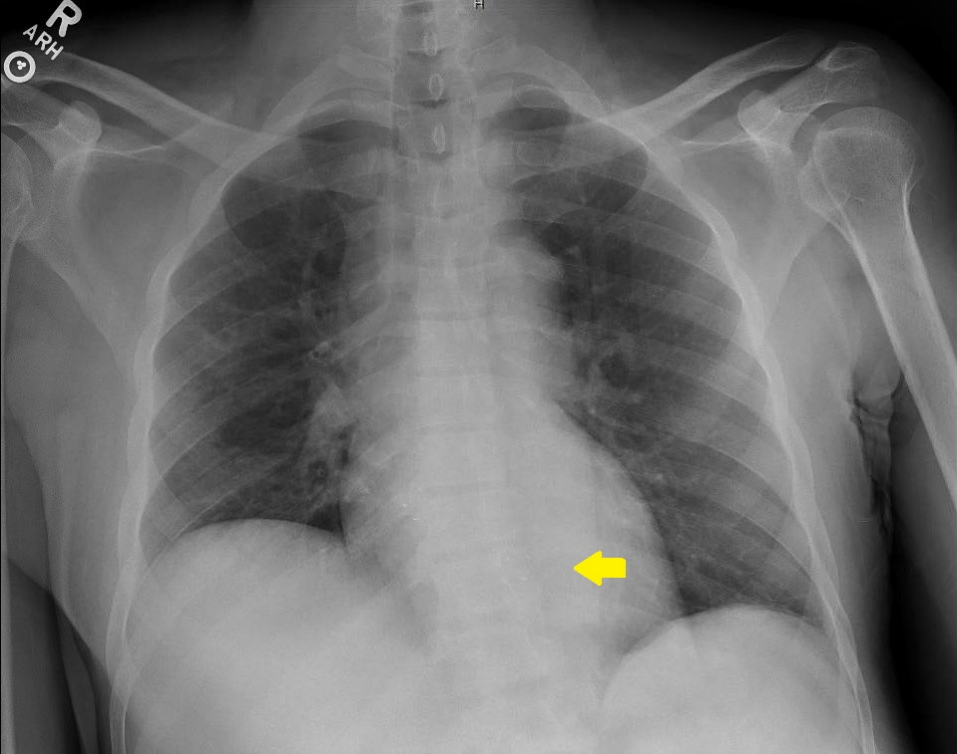
Anteroposterior chest x-ray showing punctate intracardiac markings.

**Figure 2:**
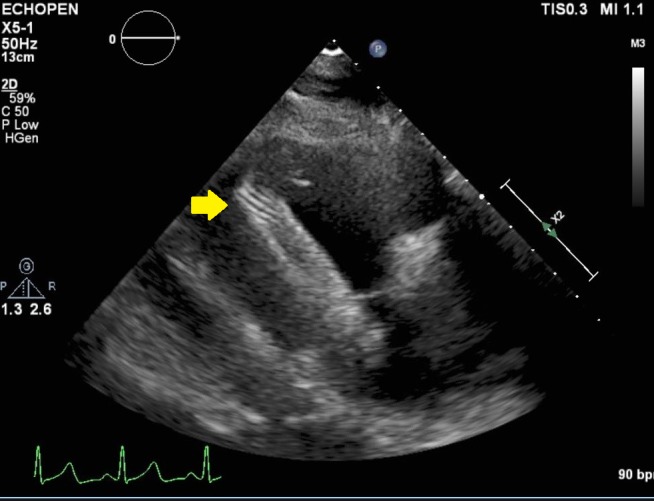
Right ventricular inflow view on trans-thoracic echocardiogram showing intracardiac stent in right ventricle.

A 61-year-old man with hypertension, end-stage kidney disease on hemodialysis (HD) via right femoral AVG, atrial fibrillation, and multiple strokes presented with impaired flow through his right thigh AVG. Previous attempts to create and maintain upper extremity vascular access failed and considered futile. Contrast fluoroscopy showed that the inflow and body of his femoral AVG access was adequate. The outflow showed two right iliac vein stenoses. Significant recoiling after angioplasty required deployment of a proximal 10 mm x 60 mm LifeStar® stent (BARD Peripheral Vascular Inc., Tempe, AZ) and a distal 10 mm x 40 mm Protégé stent (ev3 Endovascular Inc., Plymouth, MN). Three days later, the patient presented with fever, subtle punctate opacities on chest x-ray (Figure 1), and Staphylococcus aureus bacteremia. Transthoracic echocardiography showed a large intracardiac foreign body in the right ventricle (Figure 2).

**Figure 3:**
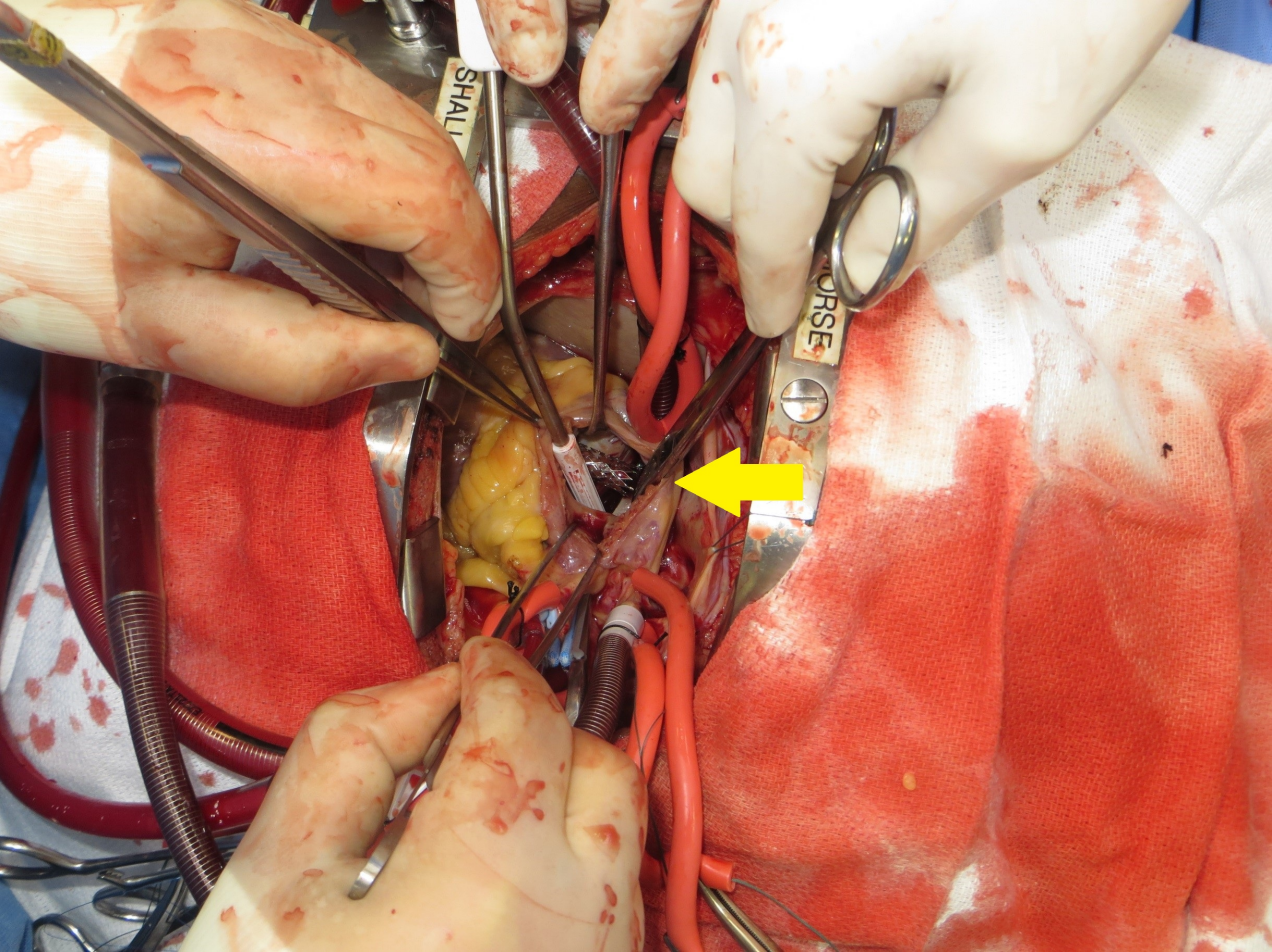
Intraoperative finding of stent in right ventricle.

An emergent cardiotomy was performed to retrieve the dislodged proximally placed stent that had migrated into the right ventricle (Figure 3). The distally placed stent in the right iliac vein was confirmed via fluoroscopy to be in adequate position. Postoperative complications were related to Staphylococcus aureus bacteremia and septic shock.

## Discussion

Patients with end-stage renal disease on HD face poor vascular access patency rates with comorbidities making them poor vascular surgery candidates. Common indications for VS placement include: significant recoiling after angioplasty, stenosis recurrence or vessel rupture [1]. Stenting provides high initial salvage rates; however, the reported primary venous patency is only approximately 20% at one year [2]. Central VSs are particularly at risk for migration given the large size of central vessels and potential vessel diameter changes associated with changes in venous return. Risk factors include variations in vessel diameter, poor wall contact, stent under-sizing, and pre-dilation of vessel segments. The use of self-expandable endovascular stents has been proposed as a method to reduce the possibility of stent migration [3]. Our patient presented Staphylococcus bacteremia, but infection as a risk factor for VS migration has not been studied or established.

## Conclusions

Stent embolization into cardiac chamber or pulmonary artery is a rare, yet serious complication to be considered following VS placement. Proposed risk factors include variations in the diameter of the vessel, poor wall contact, stent under-sizing, and pre-dilation of a pathologic vascular segment.
